# Comparative analysis of central aortic blood pressure, pulse wave velocity & arterial stiffness in patients with obstructive airway disease

**DOI:** 10.14814/phy2.16109

**Published:** 2024-09-03

**Authors:** Jyoti Bajpai, Akshyaya Pradhan, Darshan Kumar Bajaj, Ajay Kumar Verma, Surya Kant, Rishi Sethi

**Affiliations:** ^1^ Department of Respiratory Medicine King George's Medical University Lucknow Uttar Pradesh India; ^2^ Department of Cardiology King George's Medical University Lucknow India

**Keywords:** airway disease, comorbidities, pulse wave velocity, spirometry, vascular age

## Abstract

**Background:**

As the pulmonary system and cardiovascular system are intimately linked, patients with chronic obstructive pulmonary disease (COPD) and asthma have high risk for developing cardiovascular diseases (CVDs) and altered central hemodynamic.

**Objective:**

We aim to assess the central aortic blood pressure (CABP) indices, pulse wave velocity (PWV) and other indicators of arterial stiffness in Indian patients with COPD and bronchial asthma.

**Methods:**

This is a single‐center, cross‐sectional study conducted in outpatients diagnosed with either chronic stable phase of COPD or bronchial asthma. CABP indices, vascular age, arterial stiffness and central hemodynamics were measured in patients.

**Results:**

Of 193 patients with obstructive airway disease who were enrolled, (*n* = 81 had COPD and *n* = 112 had partially‐controlled bronchial asthma) the proportion of male patients was higher in both groups. The PWV, augmentation index (AI) and vascular age (VA) were significantly higher in patients with COPD compared to those with bronchial asthma (all, *p* < 0.05).

**Conclusion:**

The study showed that PWV, AI and VA were higher in patients with stable COPD without any cardiac comorbidities compared to bronchial asthma.

## 1 INTRODUCTION

Chronic respiratory diseases are among the major causes of mortality and morbidity globally. Chronic obstructive pulmonary disease (COPD) and asthma are the most common chronic respiratory diseases with a global prevalence of 3.9% and 3.6%, respectively (Labaki & Han, 2020). According to the world health organization (WHO), COPD was responsible for 3.23 million deaths worldwide in 2019 (WHO, 2021). In India, the number of COPD and asthma cases were reportedly 55.3 million and 37.9 million, respectively and both were accountable for 75.6% and 20.0% of the chronic respiratory disease disability‐adjusted life‐years (DALYs) (Salvi et al., 2018).

The lungs and cardiovascular (CV) systems are intimately linked as patients with COPD and asthma have high risk for developing cardiovascular diseases (CVDs) and altered central hemodynamics (Behnia et al., 2021; Sin & Man, 2005; Sun et al., 2018). Among chronic respiratory diseases, COPD alone accounts for 30%–50% of deaths, independently of smoking habit (Anthonisen et al., 2002). Previous studies demonstrated that reduced forced expiratory volume in 1 s (FEV1), a hallmark of COPD, was associated with ∼2‐3‐fold increase in the risk for ischemic heart disease, stroke and sudden death (Mills et al., 2008; Sin et al., 2006). In addition, reduced FEV1 was associated with increased pulse wave velocity (PWV) and individuals with reduced FEV1 are at an increased risk for developing atherosclerosis (Sin & Man, 2005). Pulmonary vascular remodeling (increased arterial stiffness [AS] occurs in COPD) consequently influences pulmonary vascular pressures (Weir‐McCall et al., 2015).

Bronchial asthma is characterized by chronic airway inflammation and bronchial hyper responsiveness. Mounting evidence reveals that adults with asthma are associated with increased risk for CVDs and all‐cause mortality (Pollevick et al., 2021; Schanen et al., 2005; Xu et al., 2017). Previous studies have elucidated that chronic inflammatory processes are likely to contribute in the pathophysiology of asthma, atherosclerosis, endothelial dysfunction, elevated PWV and vascular remodeling (Sun et al., 2014).

An increase in aortic PWV by 1 m/s corresponded to an age‐, sex‐, and risk factor‐adjusted risk increase of 14%, 15%, and 15% in total CV events, CV mortality, and all‐cause mortality, respectively (Vlachopoulos et al., 2010). Additionally, patients with COPD were more likely to be diagnosed with cardiovascular disease (odds ratio [OR] 2.46; 95% CI 2.02–3.00; *p* < 0.0001), including a two to five times higher risk of cardiovascular diseases, diseases of the pulmonary circulation, and diseases of the arteries as compared to those not having COPD (Chen et al., 2015).

Therefore, this study investigated the central aortic pressure and peripheral vascular hemodynamic variables of AS and their relationship with pulmonary function tests in obstructive (COPD and asthma).

## 2 METHODS

### 2.1 Ethics statement

We have obtained ethics approval from concerning local legislation committee before conducting this cross‐sectional study. All participants provided a written informed consent prior their enrolment, and the study was conducted by adhering to the principles of the declaration of Helsinki ethics, New drug and clinical trial rule‐2019, International Council for Harmonization‐Good Clinical Practices (ICH‐GCP) guidelines, Indian Council of Medical Research (ICMR) and the Indian GCP guidelines.

### 2.2 Study design and enrolment criteria

In this single‐centre, cross‐sectional observational study, we have recruited 193 outpatients who were diagnosed with either chronic stable phase of COPD or with bronchial asthma between January 2019 and 2020. The study was conducted at the Department of Pulmonary Medicine at King George's. Medical University in Lucknow. This study included patients with a clinical diagnosis of chronic respiratory diseases like COPD, asthma (Singh et al., 2019).

Breathlessness and tightness in the chest are common symptoms of asthma. These symptoms get worse at night or in the early morning, that they are episodic, and certain triggers (including viruses, exercise, allergies, and strong odors or cold air) might cause symptoms to flare up. COPD symptoms are comparable to those of asthma and include wheezing and dyspnea as well as a persistent cough that may or may not produce phlegm. A history of exposure to risk factors is necessary for the diagnosis of COPD, which is less common in people under 40. The most common risk factors are smoking (≥10 pack years) and biomass smoke (such as that produced by burning coal indoors for heating or cooking). Guidelines unequivocally state that pulmonary function testing (spirometry) is necessary for an asthma or COPD diagnosis. A lowered forced expiratory volume in the first second (FEV1) to forced vital capacity ratio (FVC) and an improvement in the FEV1 of at least 12% and 200 mL from the baseline value following bronchodilator administration are necessary for the diagnosis of asthma. In the case of COPD, blockage is the FEV1/FVC ratio is less than 0.7 due to a lower FEV1. Spirometry was performed while seated, with a maximum of three attempts. The diagnosis of COPD and asthma was first confirmed by spirometry, and the next day, ambulatory blood pressure monitoring was performed.

Spirometry was performed with Microquark device (from Cosmed Srl, Albano Laziale (RM), Italy). The standard American thoracic society (ATS) criteria were followed for interpretation of spirometry. The exclusion criteria was the presence of other chronic illness and conditions in patients that prevented participants from completing their participant consent form. An ambulatory blood pressure monitor Agedio B900 was used in this study. In patients with COPD and asthma, peripheral and CABP indices, vascular age (VA), AS and central hemodynamics were measured using non‐invasive Agedio B900 device (IEM, Stolberg, Germany), which works based on systolic pressure amplification phenomenon. (Pradhan et al., 2020) These indices were compared in patients with COPD and Asthma. In all patients, BP and PWV were measured in an ideal environment (sitting and quiet position). Hemoglobin (Hb) was measured by automated analyzer method.

### 2.3 Statistical analysis

Continuous variables are described as mean (standard deviation, SD) or as median and interquartile range. Categorical variables are reported as frequency (percentages). Statistical comparison was performed using the chi‐square and/student's *t*‐test. Correlation between parameters was studied using bivariate and linear regression models, and *p* < 0.05 was considered statistically significant. Statistical analysis was performed using the SPSS software version 21.0.

## 3 RESULTS

### 3.1 Demographic and baseline characteristics

In this study, 81 patients with COPD and 112 patients with bronchial asthma were enrolled. As summarized in Table 1, mean age (55.48 ± 9.97 years) of patients with COPD was significantly higher compared to the mean age of patients with asthma (48.05 ± 13.96 years, *p* < 0.001). Similar proportions of male patients were observed with asthma (61.6% [*n* = 69]) and with COPD (61.7% [*n* = 50]).

**TABLE 1:** Patient demographics and baseline characteristics.

Parameters	Bronchial asthma	COPD	*p*‐value
Age, years	48.05 ± 13.96	55.48 ± 9.97	<0.001
Gender			
Men	69 (61.6%)	50 (61.7%)	0.927
Women	43 (38.4%)	31 (38.3%)	

*Note*: Data are presented as Mean ± SD or *n* (%).

Abbreviation: COPD, chronic obstructive pulmonary disease.

### 3.2 Vascular age (VA)

Among bronchial asthma cases, around 69.6% (*n* = 78) of patients had older VA than their actual age and 24.1% (*n* = 27) of patients had younger VA than their actual age. Amid COPD cases, the proportion of older and younger VAs was observed in 70.4% (*n* = 50) and 24.7% (*n* = 20) of the patients, respectively. VA and the patient's age was similar in 6.3% and 4.9% of patients in COPD group and the asthma group, respectively. Although higher proportion of patients with COPD and asthma had VA higher/older than their actual age, no significant difference was observed in the proportion VAs between patients with COPD and asthma (*p* = 0.927) (Table 2).

**TABLE 2:** Vascular age of patients with COPD and bronchial asthma.

Vascular age	Bronchial asthma, *n* (%)	COPD, *n* (%)	Chi sq	*p*‐value
Older	78 (69.6)	57 (70.4)	0.15	0.927
Younger	27 (24.1)	20 (24.7)		
Same as actual age	7 (6.3)	4 (4.9)		

Abbreviation: COPD, chronic obstructive pulmonary disease.

### 3.3 Comparison of CABP and peripheral indices, hemodynamic variables and pulmonary parameters among COPD and asthma patients

**FIGURE 1:**
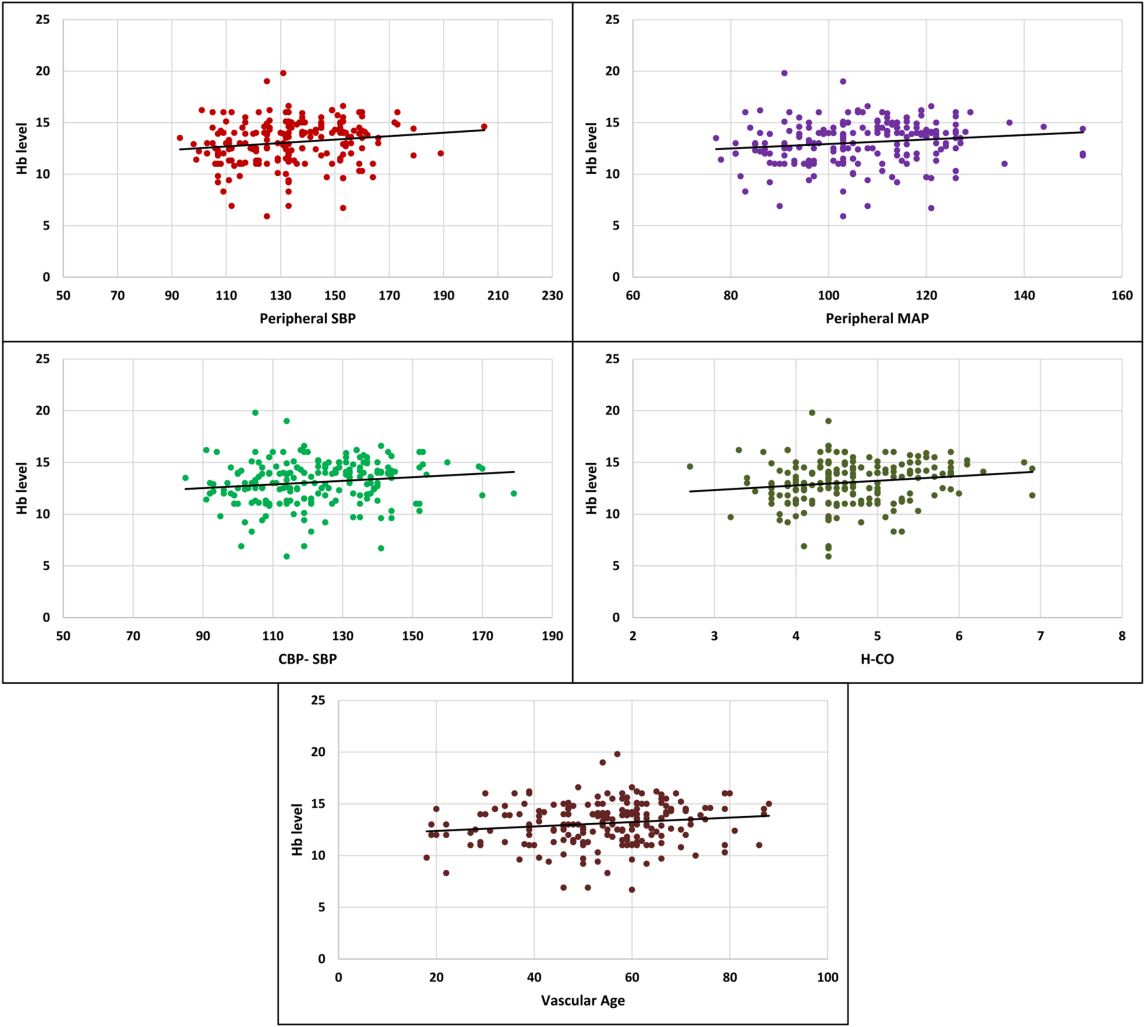
Central haemodynamic variable between patients with COPD and asthma were compared. *p < 0.05 bronchial asthma versus COPD. AI, augmentation Index; AP, augmentation pressure; CDBP, central diastolic blood pressure; cHR, circadian heart rate; COPD, chronic obstructive pulmonary disease; cPP, central perfusion pressure; cPPA, capillary pulse pressure amplitude; CSBP, central systolic blood pressure; PWV, pulse wave velocity; RC, reflection coefficient.

In patients with COPD and asthma, peripheral and CABP indices, central hemodynamic variables indicative of AS and biochemical and pulmonary function parameters were compared and summarized in Table 3. Although, the values were not statistically significant, peripheral systolic blood pressure (SBP) (asthma, 133.15 ± 18.33; COPD, 134.64 ± 22.10; *p* = 0.699), diastolic blood pressure (DBP) (asthma, 85.47 ± 10.62; COPD, 83.51 ± 15.30; *p* = 0.293) and mean arterial pressure (MAP) (asthma, 107.16 ± 13.20; COPD, 107.28 ± 15.74; *p* = 0.952) were moderately elevated in both groups (according to international guidelines and literature studies) (Melgarejo et al., 2021; Wanner & Mendes, 2010). Significant difference among both groups were observed in the following variables: AP (*p* = 0.015), AI (*p* = 0.047), PWV (*p* = 0.014) and VA (*p* < 0.001). Patients with COPD had higher AP, AI, PWV, and VA compared to patients with asthma (Figure 1).

**TABLE 3:** Peripheral and central BP indices, hemodynamic variables and pulmonary parameters in patients with COPD and asthma.

	Bronchial asthma (*n* = 112)	COPD (*n* = 81)	*p*‐value
Peripheral BP indices, mean ± SD			
SBP, mm Hg	133.51 ± 18.33	134.64 ± 22.10	0.699
DBP, mm Hg	85.47 ± 10.62	83.51 ± 15.30	0.293
MAP, mm Hg	107.16 ± 13.20	107.28 ± 15.74	0.952
PP, mm Hg	47.57 ± 12.19	50.17 ± 17.47	0.222
Central BP indices, mean ± SD			
CSBP, mm Hg	122.05 ± 15.90	123.49 ± 19.68	0.574
CDBP, mm Hg	86.94 ± 10.65	85.88 ± 13.97	0.549
cPP, mm Hg	35.05 ± 9.18	37.38 ± 14.09	0.165
cPPA	1.36 ± 0.13	1.37 ± 0.17	0.791
cHR, per min	84.10 ± 16.91	86.77 ± 15.93	0.270
AP, mm Hg	8.09 ± 5.77	10.69 ± 8.91	0.015
AI	0.27 ± 0.13	0.31 ± 0.15	0.047
RC	0.67 ± 0.08	0.67 ± 0.09	0.965
PWV	7.55 ± 1.79	9.53 ± 8.26	0.014
Hemodynamic variables, mean ± SD			
Vascular age, years	51.01 ± 15.08	58.41 ± 11.81	<0.001
H‐CO, L/min	4.67 ± 0.66	4.68 ± 0.79	0.860
H‐SV, mL	55.81 ± 8.84	54.68 ± 8.92	0.385
ABG‐pH	7.37 ± 0.08	7.37 ± 0.08	0.951
Hb mg/dL	13.04 ± 2.34	13.12 ± 1.69	0.779
TLC/mm^3^	13116.71 ± 5190.24	12484.00 ± 5949.23	0.432
TPC/mm^3^	2.19 ± 0.72	2.10 ± 0.63	0.363
Na mg/dL	137.53 ± 5.36	136.89 ± 5.63	0.421
K mg/dL	3.96 ± 1.05	3.83 ± 0.72	0.347
Urea mg/dL	42.29 ± 17.11	40.60 ± 14.08	0.468
Creatinine mg/dL	1.04 ± 0.42	1.07 ± 0.33	0.638
Serum bilirubin mg/dL	0.75 ± 0.30	0.78 ± 0.34	0.480
Pulmonary parameter, mean ± SD			
pCO_2_ (mm Hg)	53.31 ± 15.83	50.28 ± 16.58	0.199
pO_2_ (mm Hg)	60.59 ± 13.57	60.65 ± 11.80	0.974
FEV1 (L)	1.24 ± 0.50	1.13 ± 0.47	0.126
FVC (L)	2.06 ± 0.71	1.99 ± 0.73	0.459
FEV1/FVC	0.59 ± 0.09	0.57 ± 0.09	0.190

Abbreviations: ABG, arterial blood gases; AI, augmentation index; AP, augmentation pressure; BP, blood pressure; CDBP, central diastolic blood pressure; cHR, circadian heart rate; COPD, chronic obstructive pulmonary disease; cPP, central perfusion pressure; cPPA, capillary pulse pressure amplitude; CSBP, central systolic blood pressure; DBP, diastolic blood pressure; FEV, forced expiratory volume; FEV1, air exhaled during the first seconds of the forced breath; FVC, forced vital capacity; Hb, hemoglobin; H‐CO, hemodynamic cardiac output; H‐SV, hemodynamic stroke volume; L, liter; MAP, mean arterial pressure; pCO_2_, partial pressure of carbon dioxide; PP, pulse pressure; PWV, pulse wave velocity; RC, reflection coefficient; SBP, systolic blood pressure; TLC, total lung capacity; TPC, total peripheral conductance.

### 3.4 Correlation between CABP, peripheral hemodynamic variables and pulmonary parameters

**FIGURE 2:**
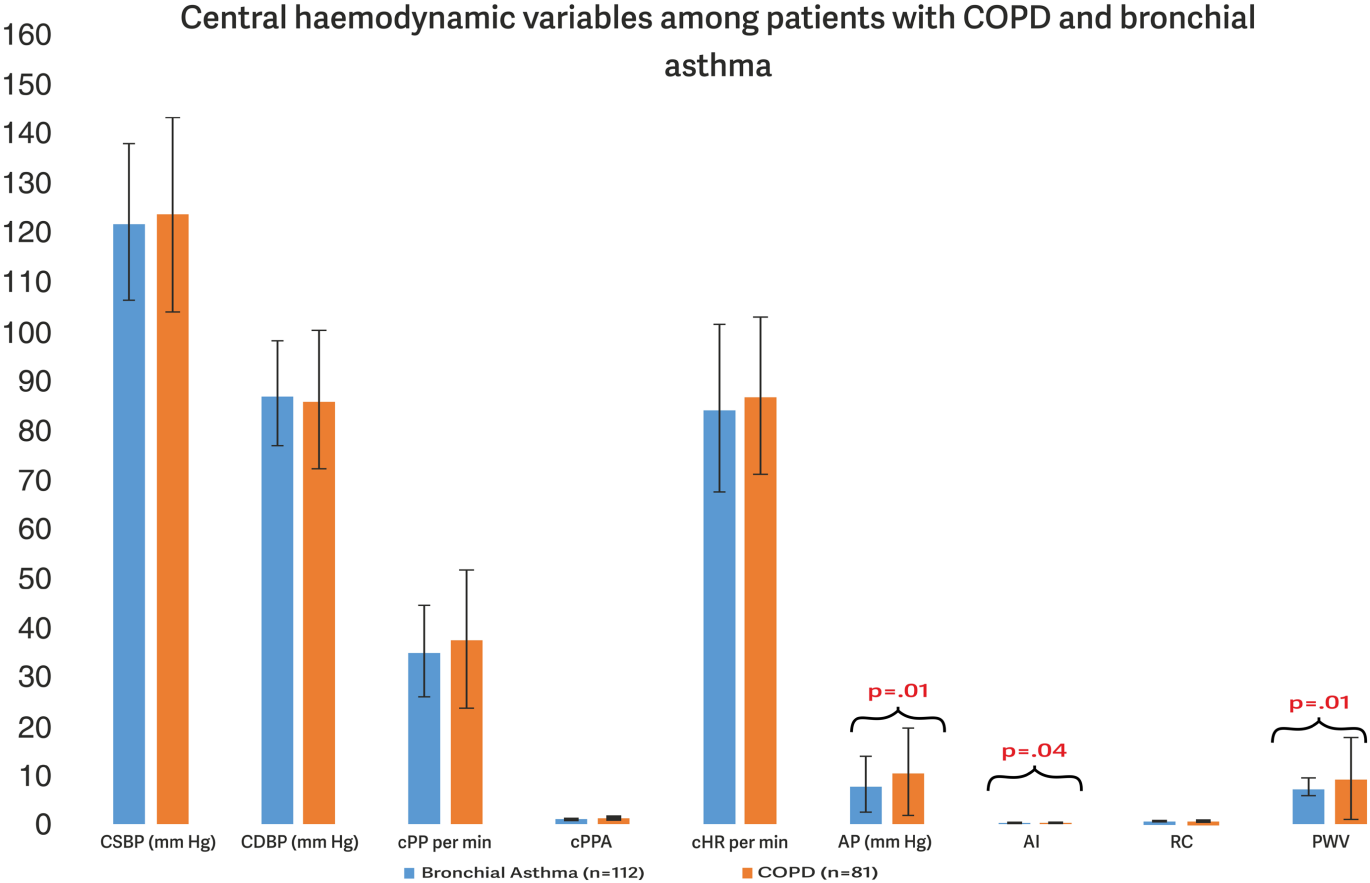
Hemoglobin showed a correlation between CABP and peripheral hemodynamic variables.

Correlation between pulmonary parameters, CABP and peripheral vascular hemodynamic variables were analyzed. Only Hb showed significant, moderate and a positive correlation with peripheral SBP (*r* = 0.156, *p* = 0.027), peripheral MAP (*r* = 0.147, *p* = 0.036), central blood pressure‐SBP (*r* = 0.145, *p* = 0.040), H‐CO (*r* = 0.151, *p* = 0.032) and VA (*r* = 0.150, *p* = 0.034). No significant association was observed between other parameters (Figure 2).

## 4 DISCUSSION

Increased burden of CVD is reported in patients with asthma and COPD (Rabe et al., 2018; Xu et al., 2017). This study investigated mechanisms of increased CV risk associated with COPD and asthma. Patients with COPD had high AP, AI, PWV, and VA compared to patients with asthma.

The AI is a component of central hypertension, an indicator of AS and an independent predictor of adverse CV events in hypertensive patients. Increased stiffness in large artery leads to elevated central aortic systolic pressures, increased left ventricular afterload and reduced diastolic coronary artery filling (Safar et al., 2003) that are important determinants of CV risk in patients with COPD. The stiffness of central and peripheral muscle arteries is reflected by PWV, which is frequently used as a measure of arterial stiffness and vascular disease. Previous studies reported that patients with COPD and asthma had higher AS Mills et al., 2008; Sabit et al., 2007; Gale et al., 2019. In this study, PWV an indicator of AS, was higher in patients with COPD patients (9.53 + 8.26; *p* = 0.014) compared to patients with asthma. In previous studies, PWV in patients with COPD patients was reportedly 11.4 ± 2.7 and 10 ± 2.4, respectively (Gale et al., 2019; Sabit et al., 2007; Safar et al., 2003).

In our study, both patients with COPD and asthma had moderately elevated SBP, DBP and MAP although the values were statistically insignificant. A previous study demonstrated that elevated SBP, DBP, MAP, and pulse pressure (PP) values contribute towards increased VA (Shimizu et al., 2019). In this study, VA of patients with COPD was higher compared to that of patients with asthma.

In this study, no significant correlation among pulmonary parameters (FEV1, FVC and FEV1/FVC), central BP indices and vascular hemodynamic variables was observed. However, Hb showed a moderately positive correlation with central hemodynamic variables. In a nation‐wide retrospective, impact of Hb levels on the risk for atrial fibrillation (AF) was analyzed. The study demonstrated that lower or higher Hb levels are associated with an increased risk for incident AF (Lim et al., 2020). Lower concentrations of Hb in COPD was associated with increased mortality risks (Toft‐Petersen et al., 2016). A cross‐sectional study, investigated the association of blood Hb with haemodynamics in 743 subjects, using whole‐body impedance cardiography and pulse wave analysis and showed that blood Hb concentration had a small direct and independent association with a measure of large artery stiffness (Choudhary et al., 2023). High Hb level has been associated with metabolic syndrome, elevated blood pressure (BP), and increased mortality risk.

### 4.1 Limitations

This study design has limitations as it was a single‐center cross‐sectional study.

### 4.2 Conclusion

The indices of vascular stiffness and central blood pressure representing the level of cardiovascular risk were considerably higher in COPD patients compared to bronchial asthma patients in patients with obstructive lung illnesses. Patients with COPD had higher PWV, augmentation pressure (AP), augmentation index (AI), and VA compared to patients with asthma. Early intervention for cardiovascular abnormalities in COPD patients is necessary to lessen morbidity and enhance patient prognosis. Our observation suggests that PWV, AP, AI, and vascular age (VA) is useful for early detection of subclinical atherosclerosis in stable COPD in comparison to stable asthma patients. Numerous studies have demonstrated that, rather than respiratory failure, cardiovascular illnesses are the main cause of death in those with mild to moderate COPD and bronchial asthma.

## AUTHOR CONTRIBUTIONS

*Concept*: Jyoti Bajpai, Akshyaya Pradhan, Rishi Sethi. *Design*: Jyoti Bajpai, Surya Kant, Ajay verma. *Definition of intellectual content*: Jyoti Bajpai, Surya Kant, Akshyaya Pradhan. *Literature search*: Jyoti Bajpai, Surya Kant, Darshan Kumar Bajaj. *Data acquisition*: Jyoti Bajpai, Surya Kant, Ajay Kumar verma. *Statistical analysis*: Jyoti Bajpai, Akshyaya Pradhan. *Manuscript preparation*: Jyoti Bajpai. *Manuscript–review & editing*: Jyoti Bajpai, Akshyaya Pradhan, Surya Kant. *Guarantor*: Jyoti Bajpai.

## FUNDING INFORMATION

The authors received no specific funding for this work.

## CONFLICT OF INTEREST STATEMENT

All authors have no conflict to disclose.

## ETHICS STATEMENT

Ethical approval from King George's Medical University Institutional Ethics Committee was taken and reference no is 96th ECM II A/P49.

## CONSENT

Written informed consent was taken.

## Data Availability

Data is within manuscript. Detailed data are available with corresponding author and will be available on request.
